# A rare twist in ohvira syndrome: When menstrual blood takes an unusual route: A case report

**DOI:** 10.1016/j.radcr.2025.08.034

**Published:** 2025-09-11

**Authors:** Rudra Prasad Ghosh, Gaurav Raj, Kaustubh Gupta, Shubhlaxmi Srivastava, Shambhavi Bisht

**Affiliations:** Department of Radio-Diagnosis, Dr. RMLIMS, Mandi Parishad Road, Vibhuti Khand, Gomti Nagar, Lucknow, Uttar Pradesh 226010, India

**Keywords:** OHVIRA syndrome, Uterus didelphys, Müllerian anomaly, Perianal fistula, Renal agenesis, Hematometra

## Abstract

Obstructed Hemivagina and Ipsilateral Renal Anomaly (OHVIRA) syndrome is a rare congenital Müllerian anomaly comprising uterus didelphys, obstructed hemivagina, and ipsilateral renal agenesis. A 20-year-old female presented with cyclical perianal bleeding and severe dysmenorrhea. Initial ultrasound revealed a large tubular cystic pelvic lesion with internal echoes, initially misinterpreted as a dilated ectopic ureter or adnexal mass, especially in the context of absent left kidney. A separate normal uterus was also visualized. On repeat ultrasound during menstruation, the lesion had decompressed via a fistulous tract opening near the perianal region, allowing recognition of the structure as a second uterine horn. MRI confirmed uterus didelphys, obstructed left hemivagina with hematometra and hematosalpinx, and a fistulous tract extending from the obstructed horn to the left perianal skin. The fistula was presumed to be an acquired complication of prior undocumented pelvic surgery. Surgical excision of the obstructed horn and fistulous tract was recommended; however, the patient declined treatment and was subsequently lost to follow-up. This case highlights a rare and diagnostically challenging variant of OHVIRA syndrome complicated by a perianal fistula, likely acquired postoperatively. It underscores the importance of considering Müllerian anomalies in adolescent females with renal agenesis and pelvic pain, especially when imaging is inconclusive. Repeat imaging timed with menses and multidisciplinary input are essential for accurate diagnosis and management.

## Background

Obstructed Hemivagina and Ipsilateral Renal Anomaly (OHVIRA) syndrome, also known as Herlyn-Werner-Wunderlich syndrome, is a rare congenital disorder characterized by a triad of Müllerian duct anomalies: uterus didelphys, an obstructed hemivagina, and ipsilateral renal agenesis [1,2]. First described in 1922, this syndrome arises from abnormal development and fusion of the paramesonephric (Müllerian) ducts, often presenting during adolescence with symptoms such as dysmenorrhea, pelvic pain, and palpable mass secondary to hematocolpos or hematometra due to vaginal obstruction [3,4]. The estimated prevalence of OHVIRA syndrome is approximately 0.1% to 3.8% in the general population, but it is likely underdiagnosed due to its variable presentation and delayed symptom onset until menarche [3].

While OHVIRA syndrome is most often associated with hematocolpos or hematometra resulting from vaginal obstruction, cases have been reported involving atypical presentations, such as febrile pediatric UTI, incomplete abortion, vaginal spotting [[5], [6], [7]]. In this report, we present a rare complication of OHVIRA — a perianal fistulous tract, which developed following prior undocumented pelvic surgery. This tract communicated with a hematosalpinx, leading to cyclical perianal bleeding. While cutaneous or perineal fistulae are not part of the classical syndrome, they may arise as iatrogenic sequelae due to improper surgical drainage or incomplete resection of the obstructed structures.

This case emphasizes the importance of early recognition of Müllerian anomalies and the need for meticulous surgical intervention to prevent chronic complications in OHVIRA syndrome.

## Case presentation

A 20-year-old woman presented to the general surgery department with complaints of cyclical blood-stained discharge from the perianal region occurring consistently during menstruation for the past 4 years. She also reported severe dysmenorrhea and chronic pelvic pain that had persisted since menarche at age 15. Her menstrual cycles were regular, but intensely painful, and associated with increased perianal bleeding and discomfort.

The patient had previously undergone multiple imaging investigations, including ultrasonography and contrast-enhanced computed tomography (CECT), which revealed left renal agenesis and a thick-walled tubular structure in the left adnexa. A provisional diagnosis of a megaureter with ectopic, dysplastic or absent left kidney was made at that time. Four years earlier, she underwent laparoscopic pelvic surgery at a different hospital, but no medical records or operative notes were available. Notably, her perianal symptoms began approximately 2–3 months postsurgery and were managed empirically by local practitioners, with no documented work-up or resolution.

**Fig. 1:**
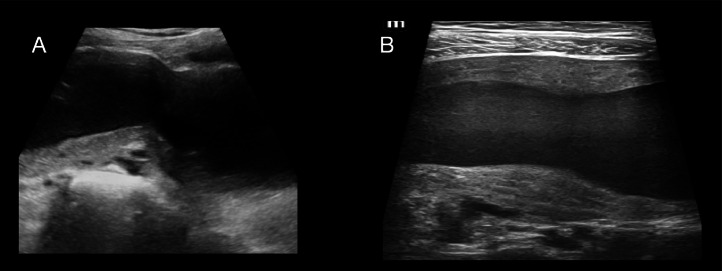
(A and B) Tubular thick walled cystic lesion in pelvis showing mobile internal echoes.

**Fig. 2:**
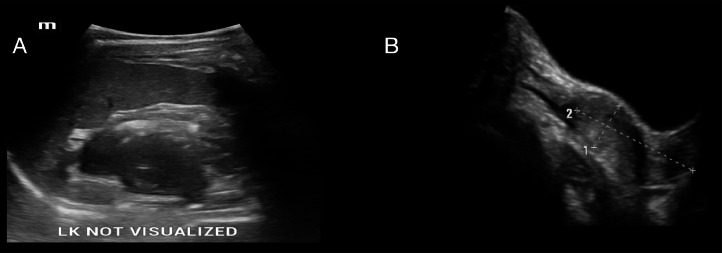
(A) Left kidney not visualised in left renal fossa and (B) Normal sized uterus noted in pelvis.

Initial pelvic ultrasound at our institution demonstrated a tubular, thick-walled cystic lesion with internal echoes in the pelvis (Fig. 1A and B). The uterus appeared normal in size and contour, and the right ovary was visualized (Fig. 2B). However, the left ovary was not identified, and the left kidney was absent from its orthotopic or common ectopic locations (Fig. 2A). A diagnosis of pelvic cystic lesion of uncertain etiology was considered, and follow-up was advised.

**Fig. 3:**
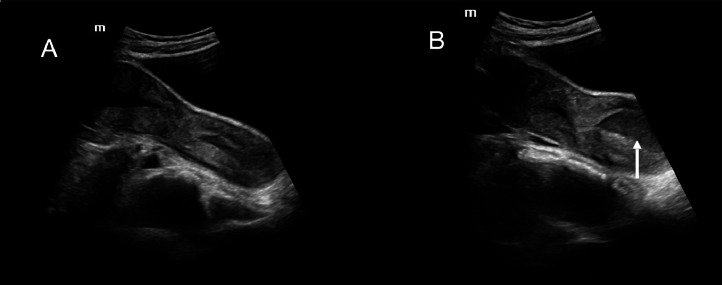
(A and B) The tubular cystic structure was identified as the second horn of a didelphic uterus. It contained fluid with mobile internal echoes suggestive of hematometra.

The patient returned during her subsequent menstrual cycle with complaints of severe dysmenorrhea and frank perianal bleeding. Repeat ultrasonography revealed a second tubular structure in the pelvis, morphologically resembling a uterus with internal fluid levels and echogenic contents suggestive of hematometra (Fig. 3A and B). A separate normally shaped uterine horn was also identified (Fig. 3, Fig. 3). These findings raised suspicion for a Müllerian anomaly, specifically uterus didelphys with an obstructed left hemivagina and hematometra. Additionally, a tubular cystic lesion with incomplete septations in the left adnexa suggested hydrosalpinx.

**Fig. 4:**
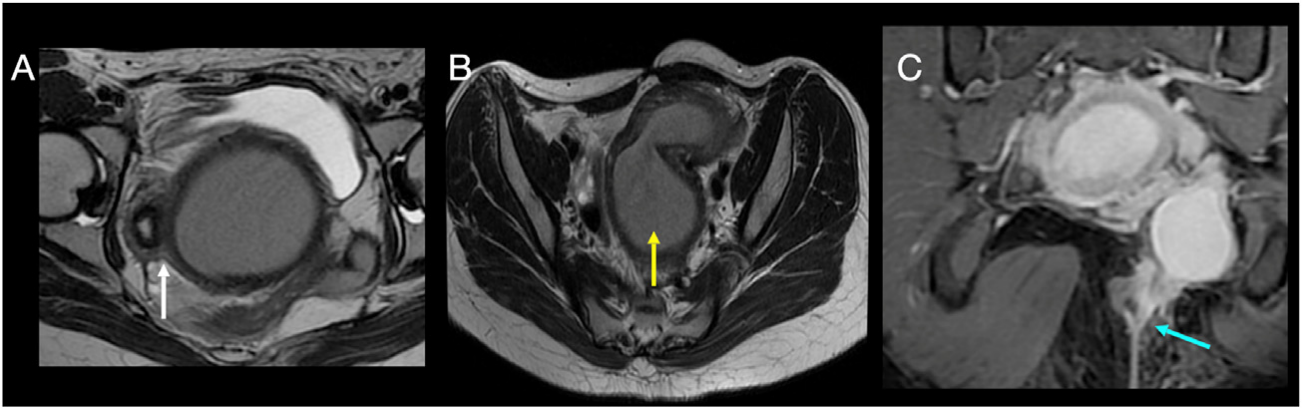
(A) T2 image showing Uterine didelphys (white arrow) with left hematometra. (B) Left hematometra (yellow arrow) and hematosalpinx and (C) Postcontrast coronal T1 GRE (LAVA) image showing enhancing fistulous tract from the hematosalpinx to the perianal skin.

To further delineate the anatomy, an MRI pelvis was performed. It revealed a thick-walled tubular structure in the pelvis containing T1 hyperintense and T2 hypointense contents, consistent with blood products (Fig. 4B). This structure was seen arising from the left vaginal region and lying adjacent to a normally developed right uterine horn (Fig. 4A). A second tubular structure, arising from the posterolateral aspect of the obstructed horn, displayed incomplete internal septations and signal characteristics of hematosalpinx. Crucially, a thin, enhancing tract was noted extending from the obstructed structure through the left ischioanal space to the perianal skin, representing a fistulous communication (Fig. 4C).

A renal DTPA scan confirmed the absence of functional renal tissue on the left side, consistent with left renal agenesis.

Based on the combination of uterine duplication, obstructed hemivagina, ipsilateral renal agenesis, and the secondary development of a perianal fistulous tract, a diagnosis of Obstructed Hemivagina and Ipsilateral Renal Anomaly (OHVIRA) syndrome with postsurgical fistulization was established.

Following the diagnosis of OHVIRA syndrome with an obstructed left hemivagina, hematometra, hematosalpinx, and a secondary perianal fistulous tract, the patient was referred for multidisciplinary evaluation involving gynecology, radiology, and colorectal surgery. Surgical excision of the obstructed structures along with fistula tract resection was recommended as the definitive treatment.

However, after counseling regarding the nature of the condition, surgical options, risks, and potential benefits, the patient declined operative intervention. She expressed preference for conservative management and opted against undergoing further surgical or invasive procedures at this time.

The patient was discharged with symptomatic treatment, including analgesics and menstrual suppression therapy, and was advised regular follow-up in the gynecology outpatient department. She was educated on potential complications such as recurrent infections, worsening of pelvic symptoms, or further fistulous complications.

## Discussion

This case highlights a diagnostically complex presentation of OHVIRA syndrome, where the classical triad of uterine didelphys, obstructed hemivagina, and ipsilateral renal agenesis was initially obscured by atypical imaging findings and an unusual clinical course. The patient’s history of prior undocumented pelvic surgery, combined with evolving symptoms and atypical ultrasound features, delayed the correct diagnosis. Notably, the presence of a perianal fistula communicating with a hematosalpinx is an extremely rare, likely iatrogenic complication, underscoring the importance of both imaging evolution and surgical history in interpreting Müllerian anomalies.

Ultrasound is often the first-line imaging modality used for pelvic complaints in adolescent girls [7]. In typical OHVIRA cases, transabdominal or transvaginal ultrasound may reveal [[1], [2], [3]]:

• Duplicated uterus with 2 separate endometrial echoes (uterus didelphys).
• Cystic mass adjacent to 1 uterine horn, representing hematocolpos or hematometra.
• Absence of 1 kidney, often on the same side as the obstructed hemivagina.
• Normal ovaries, unless distorted by associated hydrosalpinx or adhesions.

When timed appropriately during menstruation, ultrasound sensitivity improves due to the accumulation of blood products, which makes obstructed structures more visible.

In our patient, the initial ultrasound revealed a large, thick-walled tubular cystic structure with internal echoes in the pelvis. A separate normal-sized uterus with a single endometrial stripe was identified adjacent to the cystic lesion. Due to its morphology the large tubular structure was not recognized as a second uterine horn and was instead misinterpreted as a dilated ectopic ureter, adnexal mass, or a residual postsurgical pelvic collection. This misinterpretation was compounded by the nonvisualization of left ovary, and a history of previous undocumented pelvic surgery.

On repeat ultrasound during menstruation, the situation evolved in an unexpected way. Rather than showing progressive distension due to blood accumulation, the tubular structure had partially decompressed via a perianal fistulous tract. This decompression resulted in the collapsed structure assuming a more definitive uterine morphology, with identifiable endometrial lining and relation to the cervix. This led to its correct recognition as an obstructed second uterine horn, establishing the diagnosis of uterus didelphys with obstructed hemivagina.

This case underscores not only the complexity of interpreting Müllerian anomalies on ultrasound, but also how atypical decompression patterns and prior surgical interventions can mask or mimic classical features. It also reinforces the utility of repeat ultrasound with clinical correlation as a tool to reassess evolving pelvic anatomy.

MRI remains the imaging modality of choice for definitive evaluation of Müllerian anomalies. It clearly delineates uterine anatomy, vaginal obstruction, and associated complications such as hematometra or hematosalpinx [8,4]. In this case, MRI confirmed uterus didelphys, a thick-walled obstructed hemivagina, and left-sided hematosalpinx. Critically, it also identified a thin, enhancing tract traversing the ischioanal space and terminating at the perianal skin, consistent with a fistulous communication between the obstructed reproductive tract and the cutaneous surface.

The development of a perianal cutaneous fistula is not a typical manifestation of OHVIRA syndrome.Literature reports have focused on atypical presentations like febrile UTI, incomplete abortion, vaginal spotting [[5], [6], [7]]. The fistulous tract in this case most likely formed after the patient’s previous laparoscopic surgery, possibly due to unrecognized tract formation or chronic pressure effects. To our knowledge, such a complication is exceedingly rare and adds to the uniqueness of this case.

Zhu et al. proposed a classification of OHVIRA syndrome into 3 types based on communication between uterine horns and the extent of vaginal obstruction [3]. Our case most closely aligns with a variant of Type I (complete obstruction), but the presence of a perianal fistula introduces a nonclassical decompression pathway, which may merit further subclassification in future updates.

## Conclusion

This case underscores the importance of maintaining a high index of suspicion for Müllerian anomalies in adolescent females presenting with cyclical pelvic pain, dysmenorrhea, and associated renal abnormalities. Although OHVIRA syndrome typically presents with classical imaging findings, prior surgical intervention, atypical drainage pathways, or incomplete documentation can obscure diagnosis and mimic other pathologies.

In this case, the obstructed second uterine horn was misinterpreted on initial imaging, and only later identified when decompression occurred through a rare perianal fistulous tract — a complication likely related to previous surgery. This unusual presentation highlights the diagnostic challenges in complex congenital anomalies and reinforces the importance of repeat imaging, correlation with clinical symptoms, and multidisciplinary evaluation.

## Patient consent

This is to confirm that patients full consent has been attained for publication and has been explained that her diagnostic images shall be used for this academic publication for which the patient fully agrees to.
